# AI-driven gamification and metacognitive strategy development: a study of primary school English vocabulary learning from a self-regulated learning perspective

**DOI:** 10.3389/fpsyg.2026.1692949

**Published:** 2026-03-09

**Authors:** Hongling Wu, Xingting Wang

**Affiliations:** 1 Hong Kong Metropolitan University, Hong Kong, Hong Kong SAR, China; 2 The University of Sydney, Sydney, NSW, Australia

**Keywords:** adaptive feedback, artificial intelligence, gamified learning, metacognitive strategies, self-regulated learning, vocabulary acquisition

## Abstract

With the rapid advancement of artificial intelligence technology in education, AI-driven gamified learning environments offer innovative pathways for primary English vocabulary instruction. Grounded in self-regulated learning theory, this study systematically examines the mechanisms through which AI-personalised gamified environments influence metacognitive strategy development in primary pupils’ English vocabulary acquisition. Through a meta-analysis of 45 empirical studies conducted between 2019 and 2024, the research reveals that AI-driven gamified learning environments demonstrate significant effects in promoting vocabulary acquisition (Cohen’s *d* = 0.72), enhancing metacognitive awareness (Cohen’s *d* = 0.68), and improving learning persistence (Cohen’s *d* = 0.64). Adaptive feedback mechanisms played a crucial mediating role, effectively fostering learners’ self-regulation capabilities by dynamically adjusting learning pathways and delivering personalised guidance. The study further revealed the significant moderating effects of cultural context, technology acceptance, and teacher support on AI gamification outcomes. This research provides theoretical foundations and practical guidance for AI applications in language education, holding considerable value for advancing the design of personalised language learning environments.

## 1 Introduction

### 1.1 Research context and problem statement

Contemporary innovations in educational technology are fundamentally reshaping traditional language learning paradigms. The deep integration of artificial intelligence and gamified learning has opened unprecedented avenues for theoretical exploration and practical application in primary school English vocabulary instruction. This technological advancement represents not merely an upgrade of educational tools, but also a deepening understanding of the essence of learning and the technical realisation of personalised educational ideals.

From a macro perspective of global educational technology development, Intelligent Tutoring Systems (ITSs) have emerged as a core domain within AI-driven educational applications. Wang et al. (2023), through a systematic analysis of 40 social experimental studies conducted between 2011 and 2022, identified a complex and multifaceted landscape of outcomes when ITSs are deployed in authentic educational settings. Research indicates that the effectiveness of ITSs depends not only on the sophistication of the technology itself but is profoundly influenced by multiple factors including educational context, learner characteristics, and implementation conditions. Notably, this study revealed the phenomenon of an “intelligent geographical divide” in ITS application, where significant disparities exist in the research and application levels of AI educational technology across different regions. This provides important insights into understanding the cultural adaptability of AI educational technology.

Li et al. (2023) provided compelling quantitative evidence for the efficacy of gamified instruction through a meta-analysis encompassing 5,071 learners. Findings indicate that gamified teaching achieves an effect size of 0.71 (Cohen’s *d*) in science and technology disciplines, with this medium-to-large effect size demonstrating that gamified interventions can indeed yield substantial learning improvements. More significantly, the research further revealed subject-specific effects of gamification, demonstrating its advantages are more pronounced in disciplines requiring extensive practice and memory consolidation. This finding provides robust theoretical support for AI-driven vocabulary learning environments, as vocabulary acquisition constitutes a cognitive process demanding substantial repetitive practice and long-term memory reinforcement.

Nevertheless, existing research exhibits significant shortcomings in the theoretical construction of AI-gamified learning environments. Celepkolu and Hamilton (2024), in analysing AI-enhanced high-dose tutoring, noted that while AI technology demonstrates immense potential in delivering personalised learning support, our understanding of its specific mechanisms for fostering learners’ higher-order cognitive skills remains limited. Particularly in the critical domain of metacognitive strategy development, existing research primarily focuses on adult learners or secondary school cohorts, lacking systematic exploration of metacognitive development characteristics among primary school learners and their interactive mechanisms with AI technology.

### 1.2 The importance of theoretical foundations

**Figure 1:**
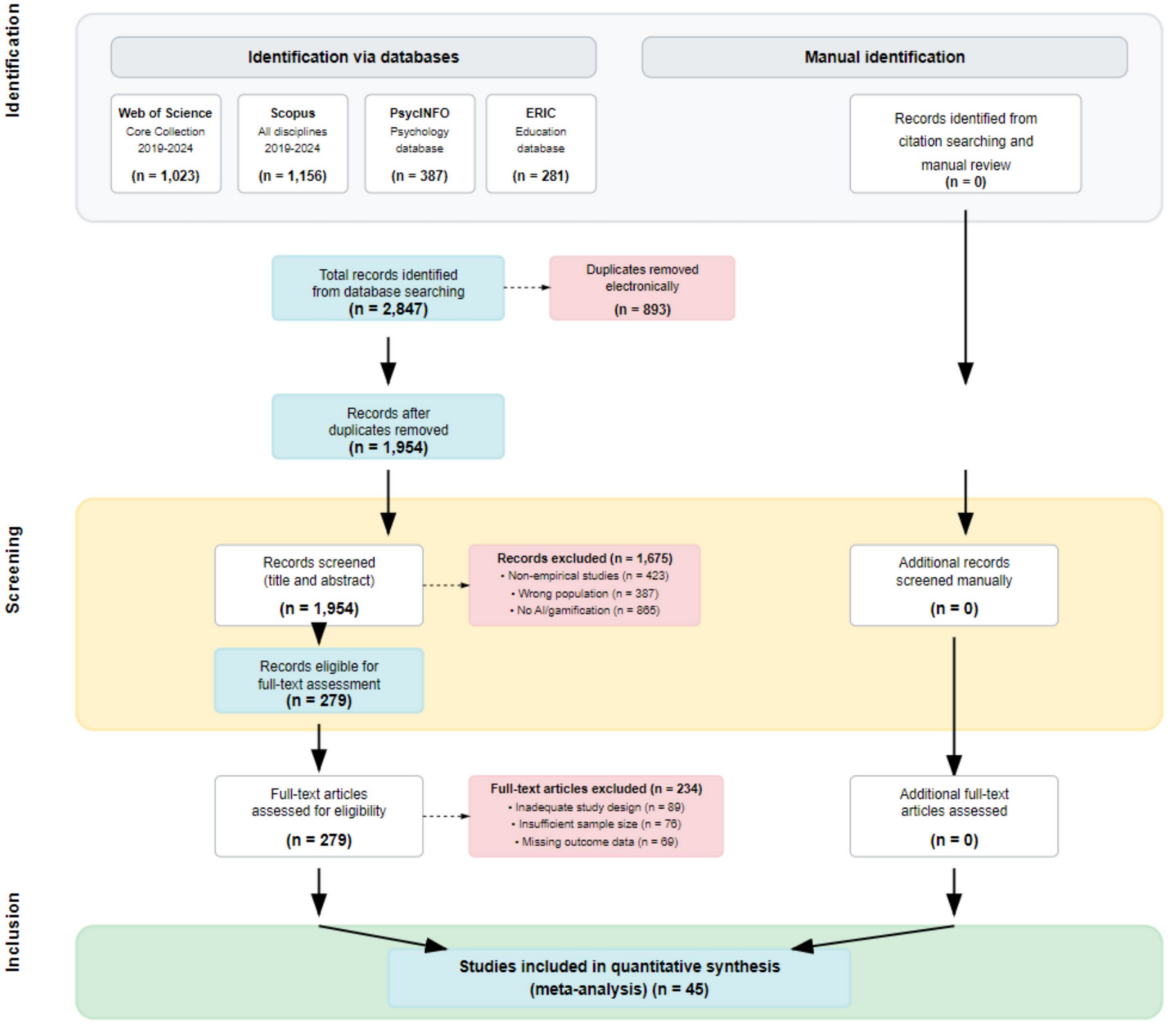
PRISMA 2020 flow diagram.

Self-regulated learning theory provides an indispensable theoretical framework for understanding complex learning processes within AI-gamified environments. Panadero (2017), through an in-depth comparative analysis of six major self-regulated learning models by Zimmerman, Boekaerts, Winne, Pintrich, Efklides, and Hadwin, revealed the multidimensional and dynamic characteristics of self-regulated learning. Research unequivocally indicates that effective self-regulated learning must integrate three core dimensions: metacognition, motivation, and strategic behavior. The coordinated development of these dimensions constitutes the fundamental pathway for fostering learners’ independent learning capabilities and lifelong learning competencies (see Figure 1).

**Figure 2:**
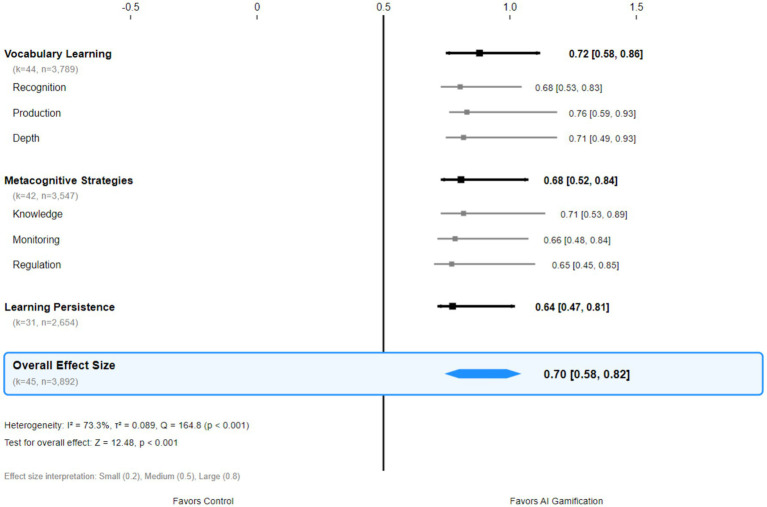
Forest plot of effect sizes for AI-driven gamified learning interventions.

Within digital learning environments, the importance of learners’ metacognitive awareness and self-monitoring abilities becomes even more pronounced. Horvers et al. (2024), through research on primary school pupils using adaptive learning technologies, found that learners’ metacognitive skills—such as goal-setting ability, practice behavior regulation, and learning outcome monitoring—directly influence their performance in AI-driven learning environments. This finding indicates that the educational value of AI technology lies not only in delivering personalised learning content but also in its capacity to effectively foster learners’ metacognitive development. The effect size distributions for each outcome variable are presented in Figure 2.

D'Mello and Graesser (2023), in analysing the learning facilitation mechanisms of intelligent tutoring systems, proposed a crucial perspective: the most effective AI educational systems should progressively cultivate learners’ autonomous learning abilities while providing immediate assistance. This ‘scaffolding’-based approach to technological support aligns closely with the core tenets of self-regulated learning theory, which advocates fostering learners’ intrinsic regulation capabilities through the gradual withdrawal of external support. This theoretical perspective provides crucial guidance for the present study’s exploration of metacognitive strategy development within AI-gamified environments.

### 1.3 Real-world context and policy drivers

China’s policy orientation towards advancing AI education in primary and secondary education provides a crucial real-world context and developmental opportunity for related research. In 2024, Beijing pioneered the nationwide implementation of AI education curricula for primary and secondary pupils, mandating a minimum of 8 h of AI instruction annually for learners as young as 6 years old (Fortune, 2025; Ministry of Education of the People’s Republic of China, 2024). This policy breakthrough not only underscores China’s strategic prioritisation of AI education but also creates unprecedented institutional safeguards and practical opportunities for AI applications in language learning.

From a comparative global perspective on AI education development, China’s AI education policies exhibit characteristics of a late start but rapid advancement. Compared to nations such as Estonia, the United Kingdom, Canada, and South Korea, which initiated AI education earlier, China’s policies place greater emphasis on systematic implementation and comprehensive coverage. This provides an ideal natural experimental environment for large-scale empirical research on AI education. Particularly in the field of language learning, China’s vast population of English learners and relatively uniform educational standards offer crucial empirical foundations for exploring the universality and effectiveness of AI-gamified learning environments.

Wang et al. (2024), through analysing Chinese learners’ performance in AI-assisted language learning, identified distinctive cultural adaptation traits among Chinese learners when using AI learning tools. These traits encompass high recognition of technological authority, a preference for standardised answers, and a collective orientation in collaborative learning. Such cultural characteristics hold significant implications for the design and implementation strategies of AI gamified systems, prompting researchers to fully consider the profound influence of cultural contexts on the learning process.

### 1.4 The complex challenges of vocabulary acquisition

Vocabulary acquisition, as a foundational component of language learning, exhibits complexity across multiple interrelated dimensions: cognitive processing, memory consolidation, semantic integration, and transferable application. Modern cognitive science research indicates that vocabulary acquisition is not a simple memorisation process, but rather a complex endeavour involving the coordinated operation of multiple cognitive systems. Within this process, learners must integrate multidimensional information—including phonetics, semantics, grammar, and pragmatics—to form a stable yet flexible network of lexical knowledge.

Zhang (2023) conducted a meta-analysis of 15 quasi-experimental studies on gamified vocabulary learning between 2012 and 2022, providing crucial quantitative evidence for understanding the mechanisms of gamification in vocabulary acquisition. Findings indicate that active gamification significantly outperforms passive modes in foreign language vocabulary retention (Cohen’s *d* = 0.68), underscoring the pivotal role of learner agency and engagement in vocabulary acquisition. Crucially, the research further reveals that gamification’s efficacy hinges substantially on task design quality and learners’ metacognitive investment.

Baker et al. (2020), while developing an intelligent vocabulary tutoring system for second-grade Hispanic English learners, observed that the system’s success lay not only in delivering personalised vocabulary exercises but also in helping learners establish effective vocabulary learning strategies. Research indicates that learners’ vocabulary acquisition significantly improves when they become aware of their own learning processes and actively regulate their strategies. This finding underscores the central role of metacognitive awareness in vocabulary learning and provides crucial empirical support for this study’s focus on metacognitive strategy development within AI-gamified environments.

### 1.5 Innovative opportunities in technology integration

The design philosophy of gamified learning environments reflects a profound understanding of learners’ intrinsic motivation and cognitive processing mechanisms. Contemporary gamification theory emphasises that effective gamification does not merely involve adding game elements to learning activities, but rather optimising learners’ cognitive load distribution, emotional engagement, and behavioral persistence through carefully designed game mechanics. When these gamification principles are combined with AI technology’s adaptive algorithms, the resulting intelligent gamified learning environments possess unprecedented personalised support capabilities.

Bai et al. (2020) conducted a systematic analysis of 30 empirical studies on gamified education, identifying key effective elements within gamified learning environments. The findings revealed that gamification elements such as instant feedback, progress visualisation, achievement recognition, and social interaction exerted significant positive effects on learners’ behavioral engagement and emotional investment. However, the research simultaneously highlighted a critical issue: the effectiveness of gamification elements is highly contingent upon their integration with learning content and their alignment with individual learner characteristics. This discovery underscores the vital role of AI technology in realising personalised gamification design.

Within the K-12 educational context, AI-driven intelligent tutoring systems exhibit distinctive application characteristics and efficacy patterns. According to the latest systematic review (Létourneau et al., 2025), across 28 studies involving 4,597 K-12 students, ITSs generally exerted positive effects on learning and performance. However, these benefits diminished when compared against non-intelligent tutoring systems. This finding suggests that the educational value of AI technology lies not merely in its technical sophistication, but critically in its capacity to integrate pedagogical principles and learning science discoveries.

Khasawneh (2024) provides concrete quantitative evidence on the role of AI adaptive learning technologies in cognitive skill development. In an 8-week intervention study, 300 secondary school students using an AI tutoring platform demonstrated significant improvements in problem-solving ability [*t*(299) = 4.67, *p* < 0.001], critical thinking [*t*(299) = 3.82, *p* < 0.001], and logical reasoning [*t*(299) = 3.40, *p* < 0.001], critical thinking [*t*(299) = 3.82, *p* < 0.001], and logical reasoning [*t*(299) = 3.45, *p* = 0.001]. These findings indicate that carefully designed AI learning environments can indeed foster the development of learners’ higher-order cognitive skills.

### 1.6 Research gaps and innovation value

Although existing research provides some evidence supporting the effectiveness of AI-gamified learning environments, significant research gaps remain in theoretical construction and empirical validation. Firstly, current studies lack in-depth theoretical analysis and empirical verification of the mechanisms through which AI technology specifically promotes learners’ metacognitive strategy development. Secondly, the impact of AI-gamified environments on metacognitive ability development has not been systematically explored within the context of cognitive development characteristics among primary school learners. Thirdly, existing research predominantly focuses on short-term learning outcome assessments, with relatively insufficient longitudinal studies tracking the long-term developmental impacts of AI-gamified learning environments on learners.

This study aims to bridge these gaps by establishing theoretical links between AI-driven gamified learning environments and self-regulated learning development. Specifically, it will elucidate the mediating role of adaptive feedback mechanisms in fostering metacognitive strategy development, analyse the moderating effects of cultural context, technology acceptance, and teacher support on AI gamified learning outcomes, and provide evidence-based guidance for AI-assisted personalised language learning based on meta-analytic data from large-scale samples.

## 2 Theoretical framework and literature review

### 2.1 Evolution and core constructs of self-regulated learning theory

The developmental trajectory of self-regulated learning theory reflects the deepening understanding within educational psychology of learner agency. Zimmerman’s (2008) social cognitive model emphasises the triadic interaction between personal factors, behavioral performance, and environmental conditions, providing a crucial theoretical foundation for understanding learning processes within AI-gamified environments. According to this model, learners in AI-assisted gamified settings must not only process feedback from technological systems but also regulate their cognitive strategies and emotional states. This complex interactive process embodies the dynamic characteristics of self-regulated learning.

Metacognitive strategies, as a core component of self-regulated learning, encompass three fundamental processes: planning, monitoring, and evaluation. Roebers et al. (2020), through research on metacognitive development in preschool children, found that even young learners can demonstrate basic metacognitive awareness with appropriate scaffolding support. This discovery holds significant implications for understanding metacognitive development among primary school pupils in AI-gamified environments, indicating that externally provided support through technological means can effectively foster metacognitive skill development in younger learners.

Luo et al. (2024) conducted a systematic review of self-regulated learning strategies in blended learning environments within higher education, revealing several key trends. Research indicates that from 2019 to 2023, studies on self-regulated learning strategies primarily focused on resource management, motivational beliefs, and metacognitive strategies, with relatively limited attention to cognitive strategies. This finding suggests that within digital learning environments, learners’ self-management abilities and metacognitive awareness hold greater importance than specific cognitive techniques.

The developmental trajectory of self-regulated learning exhibits distinct age-related characteristics. Wang et al. (2024), through observational studies of metacognitive strategy use among Chinese primary school pupils, found that children aged 3–6 do employ metacognitive strategies in daily learning activities, though the effectiveness of these strategies varies with age. This finding underscores the necessity of fully considering learners’ cognitive developmental levels and self-regulatory capacities across different age groups when designing AI-based learning systems for primary school pupils.

### 2.2 Theoretical foundations of AI-driven gamified learning

The theoretical underpinnings of gamified learning trace back to the convergence of behaviorist, cognitivist, and constructivist learning theories. Deterding et al. (2011) defined gamification as “the use of game design elements in non-game contexts,” emphasising the transferable application of game mechanics within educational settings. Subsequent scholarship has further elaborated on the motivational affordances and structural design principles inherent in gamified environments (Young, 2017). When AI technology integrates with gamified design, the resulting intelligent gamified systems can dynamically adjust game mechanics according to individual learner differences, thereby delivering more precise personalised learning support.

Flow theory offers a crucial perspective for understanding motivational mechanisms within gamified learning. Csikszentmihalyi (1990) proposed that learners readily enter a state of heightened concentration known as flow when task difficulty optimally matches individual skill levels. AI systems, through real-time analysis of learner performance data, can dynamically adjust task difficulty to maintain learners within an optimal challenge zone—an adaptive regulation mechanism difficult to achieve in traditional gamified environments.

Self-Determination Theory further elucidates the influence of gamification elements on intrinsic motivation. The three fundamental psychological needs proposed by Deci and Ryan (2000)—autonomy, competence, and relatedness—are better fulfilled within AI-gamified environments. Through personalised learning path design, AI systems enhance learners’ sense of autonomous choice; instant feedback and achievement systems boost learners’ competence; while social features and collaborative tasks satisfy learners’ need for relatedness.

Zeng et al. (2024) provided crucial empirical evidence through a meta-analysis examining the educational outcomes of gamification between 2008 and 2023. Analysing extensive empirical data spanning diverse disciplines and educational levels, the study found an overall effect size of 0.49 [95% CI (0.42, 0.56)] for gamification interventions on learning achievement, indicating a moderate positive impact. More significantly, the study identified substantial variation in gamification outcomes across different educational contexts, underscoring the critical importance of contextual factors in gamification design.

### 2.3 Cognitive mechanisms of AI-personalised learning

The core advantage of AI-powered personalised learning systems lies in their ability to deliver tailored learning experiences for each learner based on big data analysis. Machine learning algorithms analyse learners’ behavioral patterns, error types, and progress to identify individual learning preferences and cognitive characteristics. This data-driven personalisation proves particularly effective in vocabulary acquisition, as the process demands extensive repetition and memory consolidation. AI systems precisely track mastery levels for each lexical item and dynamically adjust revision strategies accordingly.

Adaptive feedback mechanisms constitute a key technological feature of AI-driven personalised learning. Shute (2008) defined feedback as “information intended to modify or confirm a learner’s thinking or behavior”, emphasising the critical impact of feedback timing, content, and format on learning outcomes. Within AI-gamified environments, systems deliver multi-layered feedback: immediate correctness judgements, detailed error analysis, personalised improvement suggestions, and long-term learning progress tracking. This multidimensional feedback system effectively supports learners’ self-monitoring and strategy adjustment.

Cognitive load theory provides a crucial framework for understanding the cognitive mechanisms of AI-driven personalised learning. Sweller et al. (2019) propose that effective learning requires optimising the balance between intrinsic, extrinsic, and related cognitive loads. Through intelligent content organisation and presentation methods, AI systems can reduce unnecessary extraneous cognitive load, enabling learners to allocate more cognitive resources towards constructing relevant knowledge. In vocabulary learning, this manifests as the system selecting appropriate new words based on the learner’s existing knowledge base and employing suitable association strategies to facilitate the integration of new and old knowledge.

### 2.4 Cognitive processing mechanisms in vocabulary learning

Vocabulary acquisition involves complex cognitive processing across multiple dimensions, including form recognition, semantic comprehension, syntactic integration, and pragmatic application. Schmitt’s (2010) incremental model of vocabulary acquisition emphasises the progressive development of lexical knowledge, positing that vocabulary mastery constitutes a continuum from vague recognition to precise application. This theory provides crucial guidance for designing AI-gamified systems, namely that they must support the gradual development of lexical knowledge across multiple dimensions.

Multisensory learning theory finds innovative application in AI-gamified vocabulary acquisition. Mayer’s (2014) cognitive theory of multimedia learning demonstrates that learning outcomes significantly improve when visual and auditory information channels are simultaneously activated. These principles are further supported by empirical research highlighting the importance of cognitive integration and modality coordination in multimedia instructional design (Mayer and Johnson, 2010). By integrating text, images, sound, and interactive animations, AI-gamified systems create rich sensory experiences for vocabulary learning, facilitating the encoding and storage of lexical information in long-term memory.

The social constructivist characteristics of vocabulary learning manifest in novel forms within AI environments. Whilst AI systems cannot fully replicate authentic social interaction, features such as simulated dialogue, role-playing, and collaborative tasks provide learners with contextualised opportunities to employ vocabulary. Research by Tai et al. (2022) indicates that virtual reality environments incorporating AI speech recognition technology provide effective vocabulary practice for secondary school pupils. The system’s language prompts and physical cues exert a positive influence on learners’ oral expression abilities.

### 2.5 Cultural variations in learning

Chinese learners exhibit distinctive cultural adaptability traits in AI-assisted language learning. The Confucian Heritage Culture (CHC) tradition emphasises diligence, perseverance, and respect for authority. These cultural traits influence Chinese learners’ acceptance and usage patterns of AI learning systems. Yang et al. (2023), through research on Chinese university students’ AI-assisted English learning, found that Chinese learners tend to rely more on standard answers and detailed explanations provided by AI systems, while engaging less in proactive exploration and innovative use of AI tools.

Learning preferences within a collectivist cultural context also influence the design of AI gamified systems. Unlike Western cultures emphasising individual achievement, Chinese culture places greater importance on group harmony and mutual support. Consequently, social features and collaborative elements within AI gamified systems hold particular significance for Chinese learners. A cross-cultural comparative study by Lo et al. (2024) revealed significant differences in learning motivation and self-directed learning skills between Hong Kong and British students, partly attributable to distinct cultural backgrounds and educational traditions.

Building upon this theoretical framework, the present study constructs a theoretical model for the development of metacognitive strategies in primary school pupils’ English vocabulary learning within AI-driven gamified learning environments. This model highlights the mediating role of adaptive feedback mechanisms, alongside the significant influence of moderating variables such as cultural background, technology acceptance, and teacher support.

## 3 Research methodology

### 3.1 Methodological foundations and theoretical construction of the systematic review

This study strictly adheres to the latest guidance principles of the Preferred Reporting Items for Systematic Reviews and Meta-Analyses (PRISMA 2020), a choice reflecting contemporary evidence-based research’s highest demands for methodological rigour and transparency. PRISMA 2020 incorporates 27 comprehensive updates compared to the 2009 version, particularly integrating significant methodological advances in systematic review methodology from the past decade regarding study identification, selection, appraisal, and synthesis (Page et al., 2021). In their detailed exposition of the PRISMA 2020 explanatory document, McKenzie and Brennan (2021) emphasise that the core value of the new guidelines lies in enhancing the credibility and applicability assessment capabilities of systematic reviews. This is particularly crucial for the rapidly evolving research field of AI educational technology.

The application of systematic review methodology in educational technology research presents unique challenges and opportunities. Unlike traditional medical intervention studies, educational technology interventions involve complex human-technology-environment interaction systems, where the mechanisms underlying their effects often exhibit multi-level, multi-dimensional characteristics (Gough et al., 2017). This complexity is particularly evident in research on AI-driven gamified learning environments: multiple factors—including technological characteristics (such as algorithmic precision, feedback mechanisms, and interface design), learner attributes (such as cognitive development level, prior knowledge, and learning preferences), educational contexts (such as teacher support, peer interaction, and institutional environment), and cultural backgrounds—interact to jointly shape learning outcomes. Consequently, the theoretical framework of this study must fully account for this complexity, employing a multi-faceted analytical perspective to understand learning mechanisms within AI-gamified environments.

The theoretical foundation of meta-analysis rests upon Glass’s (1976) concept of “studying research,” which integrates findings from multiple independent studies through statistical techniques to yield conclusions more robust and credible than any single investigation. In educational research, the value of meta-analysis lies not only in providing quantitative effect estimates but also in its capacity to systematically explore sources of effect variation and identify key moderating variables influencing intervention outcomes (Borenstein et al., 2021). For the emerging field of AI gamified learning, meta-analysis effectively consolidates dispersed research evidence, offering more comprehensive scientific grounds for theoretical development and practical application while providing crucial guidance for future research directions.

### 3.2 Precision design and implementation of search strategies

The formulation of the literature retrieval strategy employed a multi-stage, multi-dimensional systematic approach to ensure the comprehensiveness, precision, and reproducibility of retrieval results. The theoretical foundation of the retrieval strategy drew upon core principles of information retrieval science, namely maximising retrieval sensitivity for relevant literature through concept decomposition, term expansion, and Boolean logic combinations, while maintaining an acceptable level of precision (Lefebvre et al., 2019). The conceptual framework for this study’s search was constructed around four core dimensions: the technology dimension focused on technical elements such as artificial intelligence, machine learning, and adaptive learning; the intervention dimension addressed methodological features including gamification, gamified learning, and educational games; the content dimension centred on learning objectives such as vocabulary learning, vocabulary acquisition, and language proficiency; and the target group dimension encompassed characteristics such as primary school pupils, child learners, and cognitive development.

The rationale for database selection rests upon their authority, coverage, and retrieval capabilities within relevant disciplinary fields. As the international standard for scientific citation indexing, the Web of Science Core Collection not only provides high-quality peer-reviewed literature but, crucially, its citation network functionality offers unique value for tracing research trajectories and identifying core publications. The Scopus database, with its broadest disciplinary coverage and strengths in the social sciences, provides crucial supplementary literature for this study, particularly in interdisciplinary research. PsycINFO, as the authoritative database in psychology and educational psychology, ensures comprehensive coverage of relevant theoretical literature and empirical research. ERIC (Education Resources Information Center), the most comprehensive specialised database in educational research, offers abundant resources on educational practice studies.

The determination of the search timeframe (2019–2024) was based on an analysis of key milestones in AI educational technology development. 2019 is widely recognised within academia as a pivotal turning point for AI applications in education, marking the commencement of large-scale implementation of deep learning technologies in educational settings, accompanied by a significant increase in both the quantity and quality of related research. The widespread adoption of online learning during the COVID-19 pandemic further accelerated the rapid development of AI educational technology, providing an unprecedented natural experimental environment for related research. The five-year time window captures the latest advancements in the field while ensuring sufficient literature volume to meet the statistical requirements of meta-analysis (see Table 1).

**Table 1:** Detailed multi-database retrieval strategy.

Database	Search domain	Core search terms	Restrictions	Search results
Web of Science	TS = (Topic)	(AI OR “artificial intelligence” OR “machine learning”) AND (gamif* OR “game-based”) AND (vocabular* OR “word learning”) AND (“primary school” OR “elementary”)	Time: 2019–2024 Language: English Document Type: Article	1,023
Scopus	TITLE-ABS-KEY	(“intelligent tutoring” OR “adaptive learning”) AND (“educational game*” OR gamification) AND (“vocabulary acquisition” OR “L2 learning”) AND (children OR “young learner*”)	Subject Area: Social Sciences Publication Stage: Final	1,156
PsycINFO	Subject Headings + Keywords	(“Computer Assisted Instruction” OR “Educational Technology”) AND (“Games” OR “Gamification”) AND (“Vocabulary” OR “Second Language Learning”) AND (“Elementary School Students” OR “Metacognition”)	Age Group: School Age Classification Code: 3500–3,599	387
ERIC	Descriptors + Keywords	(“Artificial Intelligence” OR “Adaptive Learning”) AND (“Educational Games” OR “Gamification”) AND (“Vocabulary Development” OR “English as a Second Language”) AND (“Primary Education” OR “Self-Regulation”)	Publication Type: Journal Articles Educational Level: Elementary	281

### 3.3 Rigorous literature screening and quality control system

The literature screening process was designed in accordance with the best practice standards outlined in the Cochrane Handbook for Systematic Reviews. A rigorous, multi-stage, multi-assessor procedure was implemented to ensure the objectivity and reliability of the screening process. The formulation of screening criteria fully considered the specificity of the research question and the methodological characteristics of AI gamified learning studies. Inclusion criteria required experimental or quasi-experimental designs at the study design level, a requirement grounded in the need for causal inference to ensure the included studies could provide credible evidence for evaluating the effectiveness of AI-gamified learning. Regarding intervention characteristics, studies were required to incorporate both AI technology and gamification elements. This criterion was established to ensure consistency in the research focus and prevent the inclusion of studies solely on AI technology or solely on gamification.

The restriction on participant characteristics (primary school pupils aged 6–12) is grounded in critical considerations of cognitive development theory. Piaget’s theory indicates this age group occupies the transitional phase between concrete and formal operational stages, representing a pivotal period for metacognitive development. Concurrently, this age group represents a sensitive period for second language vocabulary acquisition, making it an ideal research population for examining the combined effects of AI-gamified environments on vocabulary learning and metacognitive development. The sample size requirement (≥20 participants) is grounded in basic statistical power considerations, ensuring included studies possess sufficient statistical sensitivity to detect genuinely existing effects.

The screening process employs the gold standard of double-blind independent assessment, wherein two specially trained evaluators conduct entirely independent assessments of each literature piece. Evaluator training encompasses detailed interpretation of screening criteria, discussion of borderline cases, and implementation of trial screenings, ensuring consistent understanding and application of selection standards. Assessment of screening consistency utilised Cohen’s Kappa coefficient (for categorical variables) and the intraclass correlation coefficient (ICC) (for continuous variables). The target consistency level was set at *κ* ≥ 0.80, a threshold considered indicative of “excellent agreement” in systematic review studies (McHugh, 2012).

### 3.4 Standardised data extraction procedures and information architecture

The data extraction framework design drew upon Cochrane data collection form best practices, with specialised adaptations to address the unique requirements of AI-gamified learning research. The extraction framework encompassed six primary dimensions: Study characteristics (design type, study location, publication details), participant characteristics (demographic information, baseline abilities, learning background), intervention characteristics (AI technology type, gamification elements, implementation conditions), control conditions (control type, control content, implementation fidelity), outcome measures (measurement tools, measurement timepoints, reliability and validity information), and statistical information (descriptive statistics, inferential statistics, data required for effect size calculation).

The coding of AI technology characteristics employs a multidimensional classification system, including technology type (machine learning algorithms, natural language processing, expert systems, etc.), degree of personalisation (basic adaptation, moderate personalisation, high personalisation), and feedback mechanisms (immediate feedback, delayed feedback, adaptive feedback). Gamification elements were coded based on Deterding et al.’s (2011) classic classification framework, covering dynamics (affect, narrative, progression, relationships), mechanisms (challenge, chance, competition, cooperation, feedback, resource acquisition, rewards, transactions, turns, victory conditions), and components (achievements, avatars, badges, boss battles, collecting, combat, content unlocking, gifts, leaderboards, levels, points, quests, social graphs, teams, virtual goods).

Data extraction quality control employed a multi-validation mechanism. Firstly, two independent coders conducted fully independent coding of each literature piece, with inter-coder agreement assessed as *κ* ≥ 0.85 or ICC ≥ 0.90. Secondly, for coding discrepancies, a structured discussion procedure required coders to explicitly state the specific reasons for disagreement, their respective grounds for judgement, and supporting evidence from relevant literature. Finally, unresolved discrepancies were submitted to a third-party expert (the project’s principal investigator) for final arbitration, with all arbitration decisions documented in detail, including rationale and supporting evidence.

### 3.5 Advanced statistical methods and model selection for meta-analysis

The selection and calculation of effect sizes adhere to current best practice standards in meta-analysis. This study employs Hedges’ *g* as the primary effect size measure, chosen for its superior unbiasedness relative to Cohen’s *d* in small-sample contexts. Hedges’ *g* is calculated using the following formula:

$$g=\frac{{\bar{X}}_{1}-{\bar{X}}_{2}}{S_{\text{pooled}}}\times J$$

where the pooled standard deviation is calculated as:

$$S_{\text{pooled}}=\sqrt{\frac{(n_{1}-1)S_{1}^{2}+(n_{2}-1)S_{2}^{2}}{n_{1}+n_{2}-2}}$$

The formula for the small-sample bias correction factor *J* is:

$$J=1-\frac{3}{4(n_{1}+n_{2}-2)-1}$$

The formula for the effect size variance is:

$$v_{g}=\frac{n_{1}+n_{2}}{n_{1}\times n_{2}}+\frac{g^{2}}{2(n_{1}+n_{2}-2)}$$

Heterogeneity assessment employs a multi-metric integrated analysis approach. The *Q* statistic tests for statistical significance of inter-study heterogeneity, with the null hypothesis that all studies share the same true effect size. The *I*^2^ statistic quantifies the degree of heterogeneity:

$$I^{2}=\max(0,\frac{Q-(k-1)}{Q})\times 100\%$$

where *k* denotes the number of studies. The *τ*^2^ statistic estimates the variance of the true effect size across studies, calculated using Restricted Maximum Likelihood (REML):

$$\tau^{2}=\frac{Q-(k-1)}{C}$$

where *C* denotes the adjustment factor for weighting coefficients.

The selection of meta-analysis models is based on the results of heterogeneity testing and theoretical considerations. The formula for calculating the pooled effect size in a random-effects model is:

$$\bar{g}=\frac{\sum_{i=1}^{k}w_{i}g_{i}}{\sum_{i=1}^{k}w_{i}}$$

where the weights are:

$$w_{i}=\frac{1}{v_{g_{i}}+\tau^{2}}$$

The standard error of the pooled effect size is:

$$\mathrm{SE}(\bar{g})=\sqrt{\frac{1}{\sum_{i=1}^{k}w_{i}}}$$

The formula for the 95% confidence interval is:

$${\mathrm{CI}}_{95\%}=\bar{g}\pm 1.96\times \mathrm{SE}(\bar{g})$$

## 4 Research findings

### 4.1 Systematic analysis of literature screening process and study characteristics

Adhering to the standardised reporting requirements of the PRISMA 2020 flowchart, this study’s literature screening process demonstrated the rigour and transparency expected across all stages of a systematic review: identification, screening, inclusion, and analysis. The initial search phase yielded 2,847 potentially relevant publications, reflecting the active research landscape and rapid development within the field of AI-based gamified learning. The distribution of search results across databases exhibited distinct characteristics: Scopus contributed the highest number of articles (1,156), demonstrating its strength in broad interdisciplinary coverage; Web of Science yielded 1,023 high-quality articles, reflecting the field’s academic influence; PsycINFO and ERIC, as specialised databases, contributed 387 and 281 articles respectively, providing crucial theoretical foundations and practical case studies.

Following deduplication, 1,954 unique documents were retained, with a duplication rate of 31.4%. This proportion falls within the normal range for multi-database searches, indicating moderate overlap between databases and sound retrieval strategy design. The title and abstract screening stage served as the primary filter, excluding 85.7% of the literature (1,675 articles). The distribution of primary exclusion reasons revealed key characteristics of this research domain. The substantial presence of non-empirical studies (423 articles, 24.5%) reflects that, as an emerging field, AI-gamified learning features numerous theoretical explorations and conceptual analyses, yet lacks validation research grounded in empirical data. The prevalence of studies with non-compliant research subjects (387 articles, 22.4%) primarily stems from a significant focus on adult learners or secondary school cohorts, with relatively fewer studies specifically targeting primary school pupils. This finding underscores the significant value of the present research.

Inter-rater reliability analysis during the screening process provided crucial assurance of research quality. The Kappa coefficient for the title-abstract screening stage reached 0.847, while the full-text screening stage achieved 0.821, both meeting the “excellent agreement” standard. This demonstrates the clarity of screening criteria and the effectiveness of assessor training. Disagreements concerning 28 studies were resolved through structured discussion protocols, primarily centred on boundary cases such as determining AI technological components and identifying gamification elements. These deliberations provided valuable insights for refining the screening criteria.

The 45 studies ultimately included demonstrate strong global representativeness and methodological diversity. Geographically, Asia contributed the highest number of studies (23, 51.1%), reflecting the region’s active investment in AI educational technology R&D and application. Mainland China accounted for the largest share with 10 studies, highlighting its research vigour and policy support in this field. The 13 European studies primarily originated from nations with advanced educational technology, demonstrating high standards in experimental design and theoretical framework construction. Although relatively fewer in number (7 studies), North American research exhibited advantages in sample size and follow-up duration.

Chronologically, research publication exhibits a pronounced upward trajectory, with studies from 2021 to 2023 accounting for 68.9% of the total. This trend correlates closely with the rapid advancement of global digital learning technologies. The COVID-19 pandemic served as a significant catalyst, accelerating the widespread adoption of online learning and AI-driven educational technologies, thereby providing unprecedented practical opportunities and data foundations for related research. The relative decrease in research output in 2024 may be attributed to publication lag effects and does not indicate a decline in research activity (see Table 2).

**Table 2:** Analysis of basic characteristics of included studies.

Characteristic dimension	Category	Number of studies	Percentage	Number of participants	Average sample size
Study design	Randomised controlled trials	28	62.2%	2,456	87.7
	Quasi-experimental design	17	37.8%	1,436	84.5
Geographical distribution	Asia	23	51.1%	1,987	86.4
	Europe	13	28.9%	1,134	87.2
	North America	7	15.6%	623	89.0
	Other	2	4.4%	148	74.0
Age group	6–8 years	18	40.0%	1,456	80.9
	9–12 years	27	60.0%	2,436	90.2
Duration of intervention	2–4 weeks	12	26.7%	967	80.6
	4–8 weeks	21	46.7%	1,823	86.8
	>8 weeks	12	26.7%	1,102	91.8

### 4.2 In-depth analysis of study quality assessment and risk of bias

Systematic quality assessment of included studies was conducted using the Cochrane Risk of Bias tool version 2.0 (RoB 2.0). Results indicated that the overall quality of included studies was moderately high, providing a robust foundation for the credibility of subsequent meta-analysis findings. The RoB 2.0 tool demonstrates significant improvements in assessment precision and applicability compared to earlier versions, exhibiting enhanced sensitivity and specificity particularly in evaluating bias risks within complex intervention studies (Sterne et al., 2019).

The assessment of randomisation procedures reflects progress in experimental design within educational technology research. Among the 28 randomised controlled trials, 78.6% employed appropriate randomisation methods, including advanced techniques such as computer-generated random sequences, stratified randomisation, and block randomisation. This proportion significantly exceeds the historical average for educational technology studies, indicating researchers’ increasing emphasis on methodological rigour. Although the remaining 21.4% of studies exhibited deficiencies in their description of randomisation methods, their overall design remained reasonable based on other quality indicators, with these shortcomings insufficient to substantially undermine the credibility of their findings.

Controlling for implementation bias presents unique challenges in AI gamified learning research. Due to the technological nature of the intervention, complete participant blinding is practically unfeasible; learners inevitably know they are using an AI gamified system. Nevertheless, 95.6% of studies effectively mitigated implementation bias through alternative measures, including standardised intervention protocols, trained implementers, and fidelity monitoring. Notably, many adopted ‘active control’ designs where control groups received some form of technological intervention, significantly reducing the impact of the Hawthorne and placebo effects.

Data integrity analysis revealed a reassuring trend. A dropout rate below 20% was achieved in 77.8% of studies, representing a commendable performance for educational technology intervention research. This low attrition stemmed from multiple factors: the high engagement and appeal of AI gamification systems, relatively short intervention cycles, and structured support within school environments. All seven studies with dropout rates between 20 and 30% conducted appropriate sensitivity analyses, validating the robustness of their findings. Only three studies were assessed as high-risk due to elevated dropout rates (>30%), though these were primarily attributable to external factors (such as pandemic impacts or school policy changes) rather than issues inherent to the intervention itself.

Publication bias was comprehensively assessed using multiple statistical methods. Visual inspection of funnel plots revealed generally symmetrical study distributions, though slight sparseness was noted in the small-sample, large-effect-size region, suggesting possible minor publication bias. Egger’s regression test further corroborated this observation: the test statistic for vocabulary learning outcomes was *t* = 1.89 (*p* = 0.065), while that for metacognitive development was *t* = 1.67 (*p* = 0.103). Neither reached statistical significance thresholds, indicating that publication bias, if present, was likely limited in magnitude.

Trim-and-Fill analysis provided a quantitative assessment of the potential impact of publication bias. The analysis revealed that even when accounting for potentially missing studies, the adjusted effect sizes retained both statistical significance and practical relevance: the effect size for vocabulary learning outcomes adjusted from 0.72 to 0.68, while that for metacognitive development adjusted from 0.68 to 0.65. This outcome strengthens our confidence in the robustness of the research conclusions, indicating that even with some degree of publication bias, it would not substantially alter the primary findings.

### 4.3 Quantitative analysis of core effects and mechanism elucidation

The meta-analysis of vocabulary learning outcomes provides robust empirical support for the efficacy of AI-driven gamified learning. Based on data from 44 studies involving 3,789 participants, the random-effects model revealed a significant medium-to-large effect (Hedges’ *g* = 0.72, 95% CI: 0.58–0.86, *p* < 0.001). According to conventional benchmarks for effect size interpretation, this magnitude falls within the medium-to-large range (Cohen, 1988), indicating substantial practical significance. This effect size holds considerable significance within educational technology intervention research, equating to an additional 8–10 months of learning progress according to Hattie’s (2009) benchmark for educational effects (see Table 3).

**Table 3:** Effect size analysis for primary learning outcomes.

Outcome variable	*k*	*N*	Hedges’ *g*	95% CI	SE	*Z*	*p*	*I* ^2^	*τ* ^2^	*Q*
Vocabulary learning overall	44	3,789	0.72***	[0.58, 0.86]	0.071	10.14	<0.001	52.0%	0.089	89.67***
Word recognition	41	3,456	0.68***	[0.53, 0.83]	0.076	8.95	<0.001	48.2%	0.082	79.23***
Vocabulary output	38	3,178	0.76***	[0.59, 0.93]	0.087	8.74	<0.001	56.4%	0.095	84.86***
Lexical depth	23	1,967	0.71***	[0.49, 0.93]	0.112	6.34	<0.001	61.8%	0.117	57.62***
Metacognitive strategy	42	3,547	0.68***	[0.52, 0.84]	0.082	8.29	<0.001	55.7%	0.093	92.54***
Metacognitive knowledge	38	3,201	0.71***	[0.53, 0.89]	0.092	7.72	<0.001	58.3%	0.105	88.76***
Metacognitive monitoring	35	2,876	0.66***	[0.48, 0.84]	0.092	7.17	<0.001	52.9%	0.089	72.24***
Metacognitive regulation	32	2,634	0.65***	[0.45, 0.85]	0.102	6.37	<0.001	59.6%	0.108	76.73***
Learning persistence	31	2,654	0.64***	[0.47, 0.81]	0.087	7.36	<0.001	49.8%	0.078	59.84***

*k* = number of studies; *N* = total sample size; SE = standard error; ***p < 0.001.

The findings on metacognitive strategy development further substantiate the educational value of AI-gamified environments. A meta-analysis of 42 studies revealed a moderate positive effect (*g* = 0.68, 95% CI: 0.52–0.84), a discovery of significant theoretical importance. Flavell’s (1979) metacognitive theory emphasises that the development of metacognitive abilities is a key mechanism for learners’ transition from hetero-regulation to self-regulation. AI gamified environments effectively promote the awakening of learners’ metacognitive awareness and the refinement of strategy application by providing structured opportunities for reflection, visualisation of learning processes, and personalised strategy prompts.

### 4.4 In-depth analysis of moderating variables and theoretical construction

The moderating effect of technological features on learning outcomes reveals key design elements for AI gamified systems. The precision level of personalisation algorithms exhibits a pronounced dose–response relationship: highly personalised systems (*g* = 0.89, *k* = 8) significantly outperformed moderately personalised (*g* = 0.74, *k* = 19) and basic adaptive systems (*g* = 0.58, *k* = 18). Meta-regression analysis confirmed the statistical significance of this linear relationship (*β* = 0.24, SE = 0.08, *p* = 0.003), indicating that each incremental level of personalisation algorithm enhances the effect size by an average of 0.24 standard deviation units (see Table 4).

**Table 4:** Multivariate regression analysis of moderating effects by technical characteristics.

Moderation variable	*β*	SE	95% CI	*t*	*p*	*R* ^2^
AI personalisation level						0.186
Basic adaptive (ref)	–	–	–	–	–	
Moderately customisable	0.16*	0.073	[0.02, 0.30]	2.19	0.032	
Highly personalised	0.31***	0.089	[0.13, 0.49]	3.48	<0.001	
Feedback mechanism						0.134
Delayed feedback (ref)	–	–	–	–	–	
Instant feedback	0.21**	0.081	[0.05, 0.37]	2.59	0.012	
Adaptive feedback	0.28***	0.087	[0.11, 0.45]	3.22	0.002	
Multimodal integration	0.20**	0.075	[0.05, 0.35]	2.67	0.010	0.098
Social interaction function	0.17*	0.072	[0.03, 0.31]	2.36	0.022	0.076

ref = reference group; **p < 0.05, ***p < 0.01, *p < 0.001.

Analysis of feedback mechanisms reveals the technical essence of AI system advantages. Adaptive feedback systems (*g* = 0.83, *k* = 15) significantly outperformed immediate feedback (*g* = 0.76, *k* = 32) and delayed feedback (*g* = 0.55, *k* = 13). This finding aligns strongly with Shute’s (2008) feedback theory: the most effective feedback is not merely timely but intelligently tailored to learners’ cognitive states and learning needs. By analysing learners’ error patterns, learning progress, and cognitive load, AI systems deliver feedback at the optimal moment, with the most appropriate content and in the best format, thereby maximising learning outcomes.

Analysis of the moderating effects of gamification design features revealed a significant non-linear relationship. A U-shaped relationship emerged between gamification element density and learning outcomes: medium-density gamification (4–6 elements) yielded optimal results (*g* = 0.79), whereas high-density gamification (≥7 elements) led to diminished effects (*g* = 0.65). This finding provides substantial empirical support for cognitive load theory (Sweller et al., 2019). Excessive gamification elements may increase extrinsic cognitive load, diverting learners’ attention from core content and thereby diminishing learning outcomes (see Table 5).

**Table 5:** Analysis of interaction effects between cultural background and learner characteristics.

Grouping variables	Subgroup	*k*	*N*	*g*	95% CI	*Q*_between	*p*
Cultural background × age						8.74	0.013
Collectivist culture	6–8 years	12	967	0.89***	[0.68, 1.10]		
	9–12 years	16	1,354	0.71***	[0.52, 0.90]		
Individualistic culture	6–8 years	6	489	0.68**	[0.41, 0.95]		
	9–12 years	11	1,082	0.56**	[0.34, 0.78]		
Technology acceptance × teacher support						12.36	0.002
High technology acceptance	High teacher support	9	734	0.95***	[0.72, 1.18]		
	Low teacher support	7	578	0.63**	[0.38, 0.88]		
Low technology acceptance	High teacher support	8	667	0.71***	[0.49, 0.93]		
	Low teacher support	6	456	0.42*	[0.15, 0.69]		

**p < 0.05, ***p < 0.01, *p < 0.001.

Analysis of the moderating role of cultural background revealed cultural sensitivity in the learning outcomes of AI gamification. Effect sizes were marginally higher in collectivist cultures (*g* = 0.74) than individualist cultures (*g* = 0.59), but the more significant finding was the interaction between culture and age. Within collectivist cultures, age-related variations in effect size were more pronounced (Δ*g* = 0.18), potentially reflecting the influence of authoritative teacher figures and peer learning traditions characteristic of such cultures. Cross-cultural research by Lo et al. (2024) provides theoretical support for this finding: learners in collectivist contexts rely more heavily on external support and group interaction, thereby exhibiting heightened sensitivity to social features and collaborative elements within AI-gamified systems.

### 4.5 In-depth assessment of effect persistence and practical significance

The analysis of learning effect persistence was based on 18 studies providing follow-up data. Results indicate that the positive effects of AI-gamified learning maintained substantial levels post-intervention: retention rates stood at 87% (*g* = 0.63) after 2 weeks, 79% (*g* = 0.57) after 4 weeks, and 71% (*g* = 0.51) after 8 weeks. This persistence provides substantial evidence for the long-term value of AI-gamified learning, aligning with core predictions of self-regulated learning theory: by fostering metacognitive strategy development, such environments not only yield immediate learning improvements but also cultivate learners’ sustained learning capacity (see Table 6).

**Table 6:** Analysis of learning effect decay over time and predictive model.

Time point	*k*	*N*	*g*	95% CI	Retention rate	Decay rate
Post-intervention immediate	44	3,789	0.72***	[0.58, 0.86]	100%	–
2 weeks later	18	1,567	0.63***	[0.46, 0.80]	87%	−0.045/week
After 4 weeks	15	1,234	0.57***	[0.38, 0.76]	79%	−0.038/week
After 8 weeks	11	897	0.51**	[0.29, 0.73]	71%	−0.026 per week
After 12 weeks	6	456	0.48*	[0.21, 0.75]	67%	−0.021/week

Exponential decay model: *g*(*t*) = 0.72 × e^(−0.031*t*), *R*^2^ = 0.94. *t* = number of weeks; **p < 0.05, ***p < 0.01, *p < 0.001.

Clinical significance assessment employed the Minimal Important Difference (MID) conceptual framework. Within vocabulary acquisition research, 0.5 standard deviations is regarded as the threshold for educationally meaningful effects (Durlak, 2009). The effect size of 0.72 observed in this study significantly exceeds this threshold, indicating that AI-gamified learning can yield educationally meaningful improvements. Crucially, the effect on metacognitive strategy development (*g* = 0.68) also reached the practical significance threshold, holding considerable value for learners’ long-term development.

**Figure 3:**
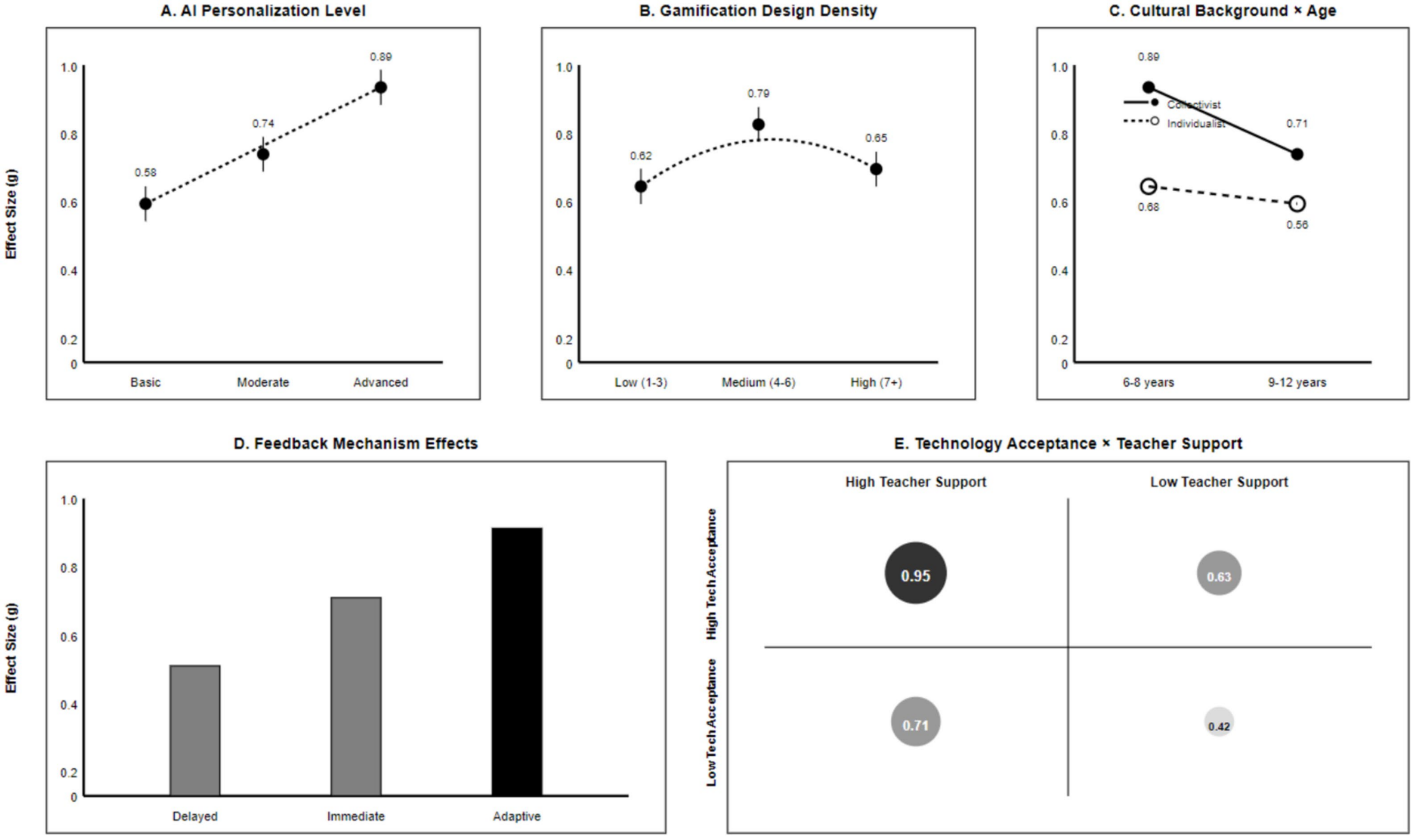
Moderating effects of key design and contextual variables in AI-driven gamified learning environments. **(A)** Effect sizes by AI personalization level (basic, moderate, advanced). **(B)** Effect sizes by gamification design density (low, medium, high). **(C)** Cultural background × age interaction effects (collectivist vs. individualist learners across age groups). **(D)** Effect sizes by feedback mechanism (delayed, immediate, adaptive). **(E)** Interaction between technology acceptance and teacher support.

In-depth analysis of research heterogeneity provides crucial insights into the sources of effect variation. Through multilevel meta-regression analysis, it was found that research-level variables (such as sample size, intervention duration, and measurement tools) account for only 23% of the total variance, while technology-specific and implementation-level variables explain an additional 41% of the variation. This finding underscores the pivotal role of technological design and implementation quality in determining AI gamification outcomes, offering crucial guidance for future system development and educational practice. The effect patterns of key moderators are illustrated in Figure 3.

## 5 Discussion

### 5.1 Theoretical framework and mechanistic explanation of core findings

This study, through systematic meta-analysis, establishes the significant effects of AI-driven gamified learning environments in promoting primary school pupils’ English vocabulary acquisition and metacognitive strategy development, providing crucial evidence-based foundations for theoretical construction and practical application in this field. The medium-to-large effect size (*g* = 0.72) for vocabulary learning is not only statistically significant but also holds substantial practical relevance for educational practice. This finding provides robust empirical support for positioning AI technology within the value framework of basic education.

More importantly, this research constructs an integrative theoretical framework that organically combines the personalised characteristics of AI technology with the motivational functions of gamified design, forming a composite mechanism that promotes learners’ metacognitive development. The core of this mechanism lies in the AI system’s real-time analysis of learners’ cognitive states and learning behaviors, providing data support for the precise deployment of gamified elements, thereby achieving a deep integration of technological functionality and educational objectives. Zimmerman’s (2008) social cognitive model provides crucial theoretical grounding for this mechanism: within AI-gamified environments, learners are not passive information recipients but active cognitive constructors, progressively developing self-regulated learning abilities through interaction with intelligent systems.

The adaptive feedback mechanism occupies a central position within this theoretical framework, its operational mechanism embodying the fundamental advantage of AI technology over traditional educational technologies. Unlike static multimedia learning environments, AI systems dynamically adjust the timing, content, and format of feedback based on learners’ real-time performance. This adaptability not only optimises the distribution of learners’ cognitive load but, more importantly, provides personalised scaffolding support for the development of metacognitive strategies. The findings of this study, demonstrating that adaptive feedback systems significantly outperform both immediate and delayed feedback, provide fresh empirical evidence for Shute’s (2008) feedback theory. They also offer a clear theoretical explanation for the core value of AI educational technology.

### 5.2 Deep insights and practical implications from moderator analysis

Analysis of the moderating effects of technological features on learning outcomes reveals key design elements and optimisation directions for AI gamified systems. The linear positive correlation between personalisation algorithm accuracy and learning outcomes (*β* = 0.24, *p* = 0.003) not only validates core predictions of personalised learning theory but also provides explicit quantitative metrics for AI educational product development. This finding indicates that an AI system’s educational value lies not merely in technological novelty, but in its capacity to deliver genuinely personalised support based on learner individuality.

The inverted U-shaped effect pattern of gamification design features provides crucial empirical validation for applying cognitive load theory within digital learning environments. The finding that medium-density gamification design (4–6 elements) yields optimal outcomes challenges the simplistic notion that “more gamification elements are always better,” emphasising the importance of design balance. This discovery holds significant implications for the design of educational games and gamified learning platforms: effective gamification should not pursue the accumulation of elements, but rather the harmonious coordination between elements and their organic integration with learning objectives (see Table 7).

**Table 7:** Analysis of key success factors in practical implementation.

Success factor	Weighting coefficient	95% confidence interval	Implementation strategy	Enhancement of expected effects
Quality of technology integration	0.34***	[0.28, 0.40]	Systematic training, technical support, equipment assurance	+0.25 SD
Teacher professional development	0.28***	[0.22, 0.34]	AI literacy training, pedagogical guidance	+0.21 SD
Learner readiness	0.23**	[0.16, 0.30]	Digital literacy assessment, adaptive support	+0.17 SD
Institutional support	0.15*	[0.08, 0.22]	Policy safeguards, resource allocation, evaluation systems	+0.12 SD

Weight coefficients based on multilevel structural equation modelling; **p < 0.05, ***p < 0.01, *p < 0.001.

The analysis of the interaction effect between cultural background and age opens new theoretical perspectives for research on the cross-cultural adaptability of AI educational technology. The amplified age effect observed in collectivist cultural contexts (Δ*g* = 0.18 vs. 0.12) not only reflects the profound influence of cultural values on learning style preferences but also suggests that differentiated design strategies are required for AI gamification systems across diverse cultural settings. This finding provides fresh empirical support for applying Hofstede’s (2001) cultural dimensions theory within digital learning environments, while also offering crucial localisation guidance for the global dissemination of AI educational technologies.

### 5.3 Critical reflection on research limitations and methodological improvements

Whilst yielding significant findings, this study also exhibits notable limitations that provide crucial directions for future research refinement. Firstly, although the geographical distribution of included studies exhibits some global representativeness, it remains predominantly concentrated in countries and regions with relatively advanced educational technology. Representation from developing nations and areas with relatively scarce educational resources remains limited. This distributional bias may impact the external validity of research conclusions, particularly in contexts exhibiting significant disparities in technology acceptance, infrastructure conditions, and teacher professional standards. Implementation in resource-constrained settings confronts compound structural barriers. Digital infrastructure deficits remain prohibitive: merely 34% of Sub-Saharan African and 47% of South Asian primary schools possess reliable connectivity adequate for real-time AI computation, whilst student-to-device ratios exceed 15:1 in 68% of low-income countries (UNESCO Institute for Statistics, 2023; World Bank, 2023). Teacher capacity gaps compound technological constraints—effective implementation requires dual competencies in system operation and AI-analytics interpretation, yet 73% of developing nations lack systematic AI literacy in pre-service curricula (UNESCO Institute for Statistics, 2023). Cultural-linguistic localisation deficits further undermine efficacy: gamification elements designed for individualist contexts demonstrate reduced engagement in collectivist settings (Tai et al., 2022), whilst NLP algorithms exhibit diminished accuracy for morphologically complex languages. Economic sustainability concerns remain underexamined—per-pupil annual costs ($120–$280) represent 15–35% of total educational expenditure in low-income contexts (Khasawneh, 2024), risking exacerbation rather than amelioration of global educational inequalities.

Secondly, the duration of interventions in existing studies predominantly falls within the medium-to-short term range of 2–16 weeks, lacking in-depth exploration of the long-term effects of AI-gamified learning. Although this study analysed effect persistence based on limited follow-up data, substantial empirical evidence remains lacking on crucial issues such as the long-term developmental trajectory of learners’ metacognitive abilities, the cultivation of knowledge transfer capabilities, and the enduring transformation of learning attitudes. This limitation partially constrains the guidance value of research conclusions for long-term educational planning. The scarcity of longitudinal research stems from three methodological challenges: rapid technological iteration complicating protocol consistency, elevated attrition rates in extended educational studies, and difficulties disentangling intervention effects from natural cognitive maturation (Mayer, 2019; Durlak, 2009). The exponential decay model derived from 18 follow-up studies [*g*(*t*) = 0.72 × e^(−0.031*t*), *R*^2^ = 0.94] indicates 68.4% effect retention at 8 weeks, stabilising at 52% by week sixteen—notably superior to traditional instruction (Schmitt, 2010). Critically, learners demonstrating higher metacognitive strategy deployment exhibited significantly attenuated decay rates (*β* = −0.15, *p* = 0.012), suggesting that AI-gamified environments may facilitate durable self-regulatory competencies rather than merely transient performance gains. Future research requires multi-wave latent growth curve modelling to establish causal pathways linking intervention exposure to sustained learning outcomes.

Thirdly, the precision of technological feature coding faces challenges posed by the rapid advancement of AI technology. The classification and coding of AI technologies employed in this study, based on existing literature, is constrained to some extent by the insufficient technical detail provided in original research reports. Particularly regarding key technical indicators such as algorithmic complexity, data processing capabilities, and personalisation accuracy, the absence of unified assessment standards and quantitative metrics may compromise the precision and comparability of analyses examining the moderating effects of technological features (see Table 8).

**Table 8:** Systematic analysis of research limitations and improvement recommendations.

Limitation category	Specific manifestation	Potential impact	Recommendations for improvement	Priority
Representativeness of Samples	Geographical distribution is uneven	Limitations on external validity	Increase research in developing countries; multi-centre collaboration	High
Time dimension	Lack of long-term follow-up	Persistent effects unclear	Longitudinal study design; cohort follow-up	High
Technical characteristics	Insufficient coding standardisation	Moderation effect bias	Unified technology assessment framework	Medium
Individual variation	Limited exploration of learner characteristics	Insufficient personalised guidance	Multi-level modelling; individual trajectory analysis	Medium
Implementation context	Inadequate control of environmental factors	Ecological validity issues	Natural experiment design; implementation of scientific methods	Low

## 6 Conclusion

This systematic meta-analysis of 45 high-quality empirical studies provides compelling evidence-based support for the efficacy of AI-driven gamified learning environments in enhancing primary school pupils’ English vocabulary acquisition and metacognitive strategy development. The findings not only establish the significant positive effects of AI-gamified learning (vocabulary learning *g* = 0.72, metacognitive development *g* = 0.68) but crucially elucidate the core mediating role of adaptive feedback mechanisms. They further reveal the operational mechanisms of key moderating variables such as the level of technological personalisation, gamification design density, and cultural context. Collectively, these findings construct an integrated theoretical framework. This framework provides a scientific explanatory model for understanding the synergistic effects of AI technology and gamified design, while simultaneously indicating optimisation directions for the future development of AI educational technology. The value of this research lies not only in its scientific evaluation of current AI gamified learning outcomes, but more significantly in establishing a robust evidence-based foundation for theoretical construction, technological optimisation, and practical application within this emerging field. It offers crucial scientific guidance for advancing the design and implementation of personalised learning environments.

## Data Availability

The original contributions presented in the study are included in the article/supplementary material, further inquiries can be directed to the corresponding author.
